# Comparison of Remifentanil and Fentanyl Regarding Hemodynamic Changes Due to Endotracheal Intubation in Preeclamptic Parturient Candidate for Cesarean Delivery

**DOI:** 10.5812/aapm.6884

**Published:** 2012-09-13

**Authors:** Alireza Pournajafian, Faranak Rokhtabnak, Alireza Kholdbarin, Mohammadreza Ghodrati, Siamak Ghavam

**Affiliations:** 1 Department of Anesthesiology, Firoozgar Hospital, Iran University of Medical Sciences (IUMS), Tehran, IR Iran

**Keywords:** Cesarean Section, Pre-Eclampsia, Intubation, Intratracheal, Fentanyl, Remifentanil

## Abstract

**Background:**

Intravenous opioids are administered to prevent and control hemodynamic changes due to endotracheal intubation. Except for special cases such as preeclampsia, these drugs are not recommended for parturants candidate for cesarean section because of the respiratory depression caused in the newborn.

**Objectives:**

According to rapid metabolism of remifentanil, the current study aimed to compare hemodynamic changes in preeclamptic parturants who received remifentanil and fentanyl for cesarean section under general anesthesia.

**Patients and Methods:**

This single blind randomized clinical trial was performed on preeclamptic pregnant women candidate for cesarean section under general anesthesia. They were divided into two groups. In the first group 0.05 μg/kg/min remifentanil was infused for 3 minutes before induction of anesthesia and in the second group 1ml (50 μg) fentanyl was injected before induction. Heart rate (HR), systolic blood pressure (SBP) and diastolic blood pressure (DBP) before and after intubation and also Apgar index were measured and compared between the two groups.

**Results:**

All hemodynamic variables increased after intubation in the fentanyl group (pSBP = 0.146, pDBP = 0.019, pHR < 0.001). Additionally, decrease in SBP (P = 0.018) and DBP (P = 0.955) and mild increase in HR (P = 0.069) after intubation in the remifentanil group was observed. No significant difference was found between Apgar indexes of the two groups (P = 0.771).

**Conclusions:**

It can be postulated that remifentanil can be used in partituents candidate for cesarean delivery under general anesthesia to prevent severe increase in blood pressure and heart rate during tracheal intubation without adverse effects on newborn.

## 1. Background

During general anesthesia, endotracheal intubation (ETI) results in stimulation of sympathetic nervous system and catecholamine release and therefore, increase in blood pressure, heart rate (HR) and heart load (1). Thus, opioids have been used to minimize these responses to short-term stimuli such as ETI in the setting of pediatrics and obstetrics as well as other situations (2, 3). Opioid administration is not recommended in general anesthesia in parturients for cesarean section, because of its depressant effects on newborn. In preeclampsia, however, the sympathetic-suppressing role of opioids seems to be crucial because ETI hypertension already exists and moreover, the ETI-induced catecholamine secretion can result in fetal arrhythmia and intracranial hemorrhage. Apart from that, opioids can decrease fetal perfusion due to their suppressive impact over circulatory system by exacerbation of aortocaval compression (4). Small doses of fentanyl are acceptable in preeclamptic parturients without adverse effects on newborn (5, 6). Therefore, an agent of short onset and offset of effects such as remifentanil seems to be of great advantage. Remifentanil has unique properties especially in pharmacokinetics which alters negligibly in liver or kidney disorders and age variations. Moreover, its rapid dissipation is a prominent privilege (7, 8).

## 2. Objectives

The current study aimed to evaluate hemodynamic changes induced by ETI if remifentanil is used compared to fentanyl. The research was carried out for the first time in the setting of cesarean delivery in preeclamptic patients.

## 3. Patients and Methods

This randomized trial was carried out in Tehran University of Medical Sciences, department of Anesthesia in Firoozgar hospital, Tehran, Iran. The study was approved by the institutional committee of ethics in Tehran University of Medical Science (No: 4276) and was registered to Iranian Registry of Clinical Trials (Irct ID: IRCT201108294969N2). Written and informed consent was obtained from all patients as well. Patients within the range of 18-45 diagnosed with preeclampsia and candidate for cesarean delivery were considered as subjects of the study. Patients presenting with cardiopulmonary disorders, hyper- or hypothyroidism, history of opioid and/or alcohol misuse, drug history of sedatives, benzodiazepines, alpha and/or beta agonists, beta antagonists and patients with complicated ETI history were excluded from the study. After allocating the patients (by block randomization) into two groups, their heart rate (HR), systolic blood pressure (SBP) and diastolic blood pressure (DBP) were recorded via noninvasive technique (by “ALBORZ B5” monitor made by “Pooyandegan Rah Saadat” Co., Tehran, Iran); also, pulse oximetry was performed in supine position as well. All patients received 500ml intravenous normal saline and were preoxygenated by 6 liters per minute flow of 100% oxygen for five minutes. Thereafter, patients in group one received 50 μg fentanyl (bolus dose), and patients in group two received remifentanil infusion at 0.05 μg/kg/min for three minutes before induction and continued until intubation time. Afterward, rapid sequence induction of anesthesia was commenced with sodium thiopental at 5 mg/kg and then succinylcholine at 1.5 mg/kg (three minutes after fentanyl injection and beginning remifentanil infusion). After cricoid pressure and 60 seconds from the latest injection, ETI was performed and all patients were mechanically ventilated (tidal volume of 10 ml/kg). Maintenance of anesthesia was provided by 0.5% Halothane + 50% N2O in oxygen. The variables of HR, SBP and DBP were recorded before ETI and immediately after that and checking the right place of tube. Apgar index was calculated for each neonate at the 1st minute after delivery. SPSS software Version 11 was employed to perform chi-square and Pair t-tests for statistical analysis.

## 4. Results

47 patients were enrolled into the study, but 43 were allocated randomly in two group. Two patients in remifentanil group and three in the other were excluded because of difficulties in tracheal intubation and need to more attempts to secure airway and ventilation. Out of remaining 38 patients, 20 and 18 were studied in remifentanil and fentanyl groups respectively. Mean age of patients was 29.14 and 28.91 years, respectively (P > 0.05). Mean of gestational age of parturients in remifentanil group was 30.11 weeks and in fentanyl group was 29.78 weeks (P = 0.12). Mean of total dose of fentanyl used in the second group was 0.735 μg/kg. Table 1 summarizes the mean values of HR, SBP and DBP in remifentanil and fentanyl groups before and after ETI. These variables had increased in level after ETI in fentanyl group. Of the three variables, HR and DBP demonstrated significant difference (P < 0.001 and 0.019, respectively). In remifentanil group, however, apart from HR that increased after ETI, SBP and DBP decreased but only differences of the SBP was significant (P = 0.018). This suggests the priority of remifentanil over fentanyl in the patients. Table 2 indicates that there was no significant difference between mean Apgar indexes of neonates in the two groups. The minimum Apgar recorded in both group was 7. The number of neonates with apgar score 7, 8, 9, 10 in remifentanil group were 1, 6, 9, 4 and in Fentanyl group were 1, 7, 7, 3 respectively. No newborn in both groups needed tracheal intubation or ventilatory support.

**Table 1:** Comparison Between Remifentanil and Fentanyl Groups Regarding Hemodynamic Markers

	Remifentanil Group		Fentanyl Group	
	Mean	*P* value	Mean	*P* value
**HR**		0.069		< 0.001
Before ETI	86.85 ± 2.482		85.17 ± 3.208	
After ETI	89.20 ± 2.676		91.94 ± 2.982	
**SBP **		0.018		0.146
Before ETI	154.35 ± 5.262		149.50 ± 3.764	
After ETI	148.35 ± 4.736		153.11 ± 4.213	
**DBP **		0.955		0.019
Before ETI	88.55 ± 3.226		87.61 ± 2.707	
After ETI	88.40 ± 2.374		92.22 ± 2.644	

^Abbreviations:^ETI, endotracheal intubation; HR, heart rate; SBP, systolic blood pressure; DBP, diastolic blood pressure.

**Table 2:** Comparison Between Remifentanil and Fentanyl Groups Regarding Apgar Index in Neonates 1min After delivery

>Apgar Index	No. (Mean ± SD)	*P* value
Remifentanil group	20 (0.880 ± 0.083)	0.771
Fentanyl group	18 (0.866 ± 0.084)	0.771

## 5. Discussion

Remifentanil is known as an ultrashort effect opioid which has a half-life of 1.3 minute. This drug has a higher clearance rate than liver blood flow and significant extra-hepatic metabolism and trivial pulmonary metabolism (9). Additionally, its concentration ratio between umbilical vein and umbilical artery is 0.88 which does not result in remarkable fetal exposure due to its rapid metabolism (10). The current study attempted to compare hemodynamic changes in preeclamptic parturients at cesarean delivery after ETI and administration of remifentanil vs. fentanyl. No severe changes were observed in hemodynamic markers of mothers but superiority of remifentanil over fentanyl can be suggested. This is because of significant decrease in SBP in remifentanil group vs. significant increase in HR and DBP in fentanyl group, both after ETI.

Safety and efficacy of remifentanil have been demonstrated in some case series and randomized controlled trials (RCTs) in pregnant women. Roelants et al. used 0.05 µg/kg per minute infusion and 25 μg bolus of remifentanil and observed 5 minutes analgesia during normal vaginal delivery (11). Moreover, it has been reported by Owen et al. that 34 hours of remifentanil infusion has no noticeable adverse effect on newborn (12). Furthermore, in the study conducted by Douma et al., superiority of remifentanil over fentanyl and meperidine in labor was provided. This had reduced the need for regional anesthetic support while no change in Apgar index was observed (13). Palacio et al. also showed no negative impact upon neonates born from parturients with obstetric emergencies when remifentanil bolus dose at 1μg/kg was administered as maintenance in cesarean sections (14). Although routinely opioids are not drugs of choice in induction of general anesthesia during cesarean section, little amounts of fentanyl is administered in cases with preeclampsia, HELLP syndrome, coagulopathies and patient’s refusal of spinal anesthesia (15, 16). In such cases, however, hemodynamic responses to ETI are unavoidable. Because of transient delayed respiratory depression with higher doses of remifentanil was noted in some newborns (17-20), in the current study safe dose of this drug that had been previously used in labor, was considered. Opioids was also used in epidural space in pregnant women for cesarean section without significant difference in maternal complications and Apgar scores (21). Safavi M et al. showed that some drugs such as Nitroglycerin are effective in attenuating the pressor response to tracheal intubation in severe preeclampsia and could be used instead of opioids to control hemodynamic responses (22). Remifentanil and fentanyl were also used effectively in endotracheal intubation without use of neuromuscular blocking drugs (23, 24). Remifentanil infusion tends to faster wake up test than alfentanil in spinal fusion surgeries (25) (because of rapid metabolism) and is suggested to be used in situations that require fast effect and rapid recovery of opioid effects. In this regard, it was postulated that remifentanil may perform a prominent role because of its stabilizing impact on heart rate, blood pressure and Apgar index. Low dose remifentanil can be used safely in parturients candidate for cesarean section without noticeable adverse effects on newborn.
